# Do Bites of Kissing Bugs Cause Unexplained Allergies? Results from a Survey in Triatomine-Exposed and Unexposed Areas in Southern California

**DOI:** 10.1371/journal.pone.0044016

**Published:** 2012-08-28

**Authors:** Jan Walter, Erin Fletcher, Roba Moussaoui, Kumar Gandhi, Christiane Weirauch

**Affiliations:** 1 Department of Plant Pathology and Microbiology, University of California Riverside, Riverside, California, United States of America; 2 Department of Entomology, University of California Riverside, Riverside, California, United States of America; Royal Tropical Institute, The Netherlands

## Abstract

**Background:**

The bite of Triatominae can cause cutaneous allergic reactions and even anaphylaxis. Since the early 1980s, no population-based surveys have been done in Southern California, and none was ever carried out in inland Los Angeles or Riverside Counties.

**Objectives:**

To measure the frequency of insect sightings, bites and allergic reactions in a suburban area of eastern Los Angeles County and along with rural and urban sites in Riverside County.

**Methods:**

A door-to-door survey was done in triatomine exposed and unexposed areas. Logistic regression modeling was used for the analysis, and study participants were asked to collect insects.

**Results:**

Out of the 221 study participants in the exposed areas, 46 (20%) knew about the presence of Triatominae in their neighborhood. Fifteen (7%) persons reported triatomine sightings in their house during the month preceding the interview. Also, 15 (7%) participants reported ever being bitten by a triatomine. Ten (5%) participants collected either a *Triatoma protracta* Uhler and/or *Paratriatoma hirsuta* Barber in and around their house. Twenty-nine (13%) persons in the rural Riverside County reported symptoms compatible with allergy to triatomine bites. This was 4 times higher than in the urban control area where only 4 (3%) of 115 persons reported these symptoms. The association between living in a triatomine-exposed area and self-reported symptoms suggestive of allergies increased slightly when adjusted for the participant’s sex and the age of their house (adjusted odds ratio: 5.1, 95% confidence interval: 1.2 to 22.0). Reporting these symptoms was associated with seeing Triatominae in the neighborhood and having been bitten.

**Conclusion:**

Allergies to triatomine bites could be a significant problem in inland Southern California. Further investigations, a diagnostic test and better information of persons living in triatomine-exposed areas are needed.

## Introduction

Triatominae (kissing bugs), an assassin bugs subfamily of ∼137 species [1], [2] that are mostly restricted to the New World, are most infamous for their ability to transmit *Trypanosoma cruzi* Chagas, which is the causative agent of Chagas disease in Central and South America. It is however less well known that triatomine bites can also cause allergic reaction ranging from mild allergies to anaphylaxis and death [3].

In California, allergies to triatomine bites have been documented as early as 1894 [4]. The symptoms have been described in detail [5] and there is a number of studies that isolated potential antigens from triatomine saliva, developed diagnostic tests and even investigated the possibility of an immuno-therapy [6]–[9]. The last population-based survey done in Southern California looked at 120 inhabitants of the foothills in Santa Barbara County and found elevated levels of IgE antibodies to *Triatoma protracta* in 8 (6.7%) persons [10]. In addition, there are isolated reports from across the Southwestern United States [11]–[15] including a death in Arizona [16], as well as from elsewhere in the world [17].

Despite previous studies that suggest frequent sensitization to triatomine salvia in costal parts of California [10] and widespread occurrence of Triatominae across the Southern United States [18], there is no FDA-approved diagnostic test commercially available at this time and no immunotherapy exists. The public health impact of triatomine bites is not well documented.

Distribution maps of the three endemic kissing bug species in Southern California were recently generated by one of us (CW) based on ongoing entomological surveys and specimens deposited in Natural History Museums (http://www.discoverlife.org/for *T. protracta*, *Triatoma rubida*, and *P. hirsuta*). These data indicate that especially *T. protracta* is common in the Greater Los Angeles area and the Morongo Basin, bordering the large metropolitan areas of Los Angeles and San Diego. To better understand the potential public health risk that triatomine bites could cause, we conducted a door-to-door survey in two triatomine-exposed and an unexposed area in Southern California. The primary aim was to measure the frequency of triatomine sightings, bites, and of allergic reactions. Surprisingly, we found symptoms suggestive of triatomine allergies to be frequent in the exposed areas.

**Table 1:** Characteristics of persons participating in a door-to-door survey in selected areas in Southern California, 2009.

	Triatomine-exposed			Triatomine*-*unexposed	p-value*
	Desert	Foothills	Total		
N	116	105	221	115	
Age [mean±SD]	49±16	54±16	51±16	53±20	0.56
Sex [n (%) male]	51 (44)	39 (38)	90 (41)	70 (61)	0.0005
Ethnicity [n (%) Caucasian]	98 (84)	90 (86)	188 (85)	91 (79)	0.17
Number of persons per household [median (IQR)]	2 (2, 4)	3 (2, 4)	2 (2,3)	2 (2, 3)	0.49
Years at current residence [median (IQR)]	5 (2, 15)	16 (10, 26)	10 (3, 22)	12 (4, 27)	0.29
Age of house [median years (IQR)]	27 (11, 36)	39 (29, 47)	32 (23, 45)	59 (53, 75)	<0.0001
Any pets [n (%)]*	93 (80)	68 (65)	161 (73)	88 (77)	0.47
Cats [n (%)]	34 (29)	22 (21)	56 (25)	35 (30)	0.32
Dogs [n (%)]	70 (60)	50 (48)	120 (54)	62 (54)	0.95
Insect tight window screens [n (%)]	88 (82)	92 (91)	180 (87)	87 (85)	0.77
Outside lights at night [n (%)]	37 (32)	44 (43)	81 (37)	62 (54)	0.003

SD = standard deviation. Numbers may slightly differ from the total due to missing data. IQR = interquartile range.

* including lizards, birds, rat, rabbits, reptiles, snake, chicken, hamster, tortoise, in addition to cats and dogs.

## Methods

### Selection of the Study Sites

Our entomological surveys using UV light traps detected specimens of *T. protracta* in settlements in Glendora and Yucca Valley, California, and these two areas were selected for this study. Both sites differ markedly in their habitat. Glendora is a suburban community in the Foothills of the San Gabriel Mountains in Los Angeles County. Yucca Valley is a more rural community in the Morongo Basin in the Mojave Desert in San Bernardino County. After data collection was completed, a control area in urban Riverside was selected and surveyed. This area comprised the streets south of the city center (wood streets), stretching over the train tracks and around Central Avenue. While the northeast of the sampling area came within about 500 m of the Santa Anna River and while it bordered the small Mountain View Park, the area was otherwise largely removed from city parks and the Santa Anna River bed, which may be inhabited by woodrats (*Neotoma* spp.), the natural hosts of Triatominae in Southern California.

**Table 2:** Main outcomes by triatomine-exposed and -unexposed area (n = 336).

	Triatomine-exposed				Triatomine*-*unexposed [n (%)]	p-value*
	Desert [n (%)]	Foothills[n (%)]	Total[% (95% CI)]			
N	116	105		221	115	
Knowing that Triatominae occur in the neighborhood	41 (35)	5 (5)		20 (10, 31)	3 (3)	<0.0001
Ever seen Triatominae in the neighborhood	34 (29)	8 (8)		19 (11, 27)	0 (0)	<0.0001
Seen Triatominae in the house during the last month	15 (13)	2 (2)		8 (1, 14)	0 (0)	0.002
Ever been bitten by Triatominae	10 (9)	5 (5)		7 (2, 12)	0 (0)	0.004
Triatominae collected [n (%)]§	5 (5)	5 (5)		5 (1, 8)	n.a	n.a
Of these:						
* T. protracta* collected [n (%)]	2 (2)	5 (5)		3 (0, 6)	n.a	n.a
* P. hirsuta* collected [n (%)]	3 (3)	0 (0)		1 (0, 3)	n.a	n.a
Unexplained allergies († or #)	18 (16)	11 (10)		13 (8, 18)	4 (3)	0.005
Of these						
dermatological symptoms†	15 (13)	11 (10)		12 (7, 17)	3 (3)	0.005
waking up with systemic symptoms						
Any	10 (9)	4 (3)		6 (3, 10)	1 (1)	0.02
swollen eyes	5 (4)	1 (1)			0 (0)	
difficulty breathing	1 (1)	0 (0)			0 (0)	
Dizziness	2 (2)	3 (3)			0 (0)	
difficulty swallowing	0 (0)	0 (0)			1 (1)	
several of the above	2 (2)	0 (0)			0 (0)	

Numbers may be small than the total due to missing data. Triatomine exposed areas were surveyed in summer, triatomine unexposed in winter, which may contribute to the observed differences.

n.a. =  not applicable, since persons in the control region were not asked to collect insects.

* Comparing exposed versus non exposed populations.

† rashes, red or itchy spots on the body,

# waking up at night or the morning feeling dizzy, sick, having swollen eyes or difficulties breathing.

§ Only one specimen per insect species listed per study participant.

### Study Design

We used a cluster randomized design to select areas for our survey. Both exposed sites were limited to the main ZIP code each. In Glendora, the survey area was further restricted to areas within 1 km of the foothills, given the limited flight range of the insects of ¼ of a mile or more [19] and dense settlement is this suburban community. Using Google Earth, we identified blocks of 50 homes, which excluded apartment houses and commercial areas. In Yucca Valley, we randomly selected 10 blocks to be surveyed, oversampling by factor 3 more remote areas that we suspected to be more exposed to Triatominae. In Glendora, we surveyed all clusters except for those including gated communities, resulting in a nearly continuous survey area. In the Riverside County urban control area, a continuous area was surveyed.

**Table 3:** Comparison of persons with and without self-reported symptoms matching those typical for allergies to triatomine bites in triatomine-exposed areas (n = 221).

	Symptoms†	No symptoms	p-value
N	29	192	
Knowing that Triatominae occur in the neighborhood [n (%)]	11 (38)	35 (18)	0.01
Ever seen Triatominae in the neighborhood [n (%)]	11 (38)	31 (16)	0.005
Seen Triatominae in the house during the last month [n (%)]	5 (17)	12 (6)	0.05
Ever been bitten by Triatominae [n (%)]	6 (21)	9 (5)	0.007
Age [mean±SD]	46±16	52±16	0.07
Sex [n (%) male]	8 (28)	82 (43)	0.12
Ethnicity [n (%) Caucasian]	26 (90)	162 (84)	0.58
Number of persons per household [median (IQR)]	3 (2, 4)	2 (2, 4)	0.53
Years at current residence [median (IQR)]	6 (3, 20)	10 (3, 22)	0.37
Age of house [median years (IQR)]	32 (25, 38)	33 (22, 45)	0.84
Any pets [n (%)]	25 (86)	136 (71)	0.08
Cats [n (%)]	7 (24)	49 (26)	0.87
Dogs [n (%)]	18 (62)	102 (53)	0.37
Insect tight window screens [n (%)]	23 (85)	157 (87)	0.77
Lights at night [n (%)]	11 (38)	70 (37)	0.93
Any Triatominae collected [n (%)]	3 (10)	7 (4)	0.12
*T. protracta* collected [n (%)]	1 (3)	2 (1)	0.35
*P. hirsuta* collected [n (%)]	2 (7)	5 (3)	0.23

Tally may be slightly lower than the total due to missing data.

† rashes, red or itchy spots on the body, waking up at night or the morning feeling dizzy, sick, having swollen eyes or difficulties breathing.

### Interviews

All houses in the selected survey areas were approached by the study team once. The person answering the door was invited to participate in an interview if above 18 years of age. If minors below 18 years of age answered the door or if the first person refused participation, another person was invited to participate in the interview. We first asked whether or not persons knew that kissing bugs, also called Triatominae or cone-nosed bugs, occur in the neighborhood. We then presented them with habitus images or pinned specimens of *T. protracta* immatures and adults mixed among other endemic insects (three hemipterans including bed bugs [*Cimex lectularius, Boisea rubrolineata, Rasahus thoracicus]* and one beetle *[Prionus californicus*]) and asked them which of these insects they had seen in the neighborhood, yard or house. If Triatominae were identified, we asked about specific numbers seen in the last month, times of sightings and bites. Sightings and bites were differentiated as certain or as possible. We collected data on symptoms following bites, socio-demographic variables, and asked if the interviewee or someone else in the house has allergies due to unknown causes including rashes, red or itchy spots in the body. Study personal was explicitly trained to only include dermatological data and to exclude allergies such as hay fever. Similarly we asked if the interviewee or someone else in the house has sometimes woken up in the night or morning feeling dizzy, sick, having swollen eyes or difficulties breathing or swallowing. For the last questions, patterns and exact symptoms were recorded as well as the age and sex of the person and whether or not this was the main interview partner.

**Table 4:** Logistic regression modeling of factors associated with self-reported symptoms typical for those to triatomine bites among persons participating in the survey (n = 336).

	Unadjusted OR (95% CI)	Adjusted OR* (95% CI)
Living in triatomine exposed areas	4.2 (1.4, 12.3)	5.1 (1.2, 22.0)
Sex [male versus female]	0.4 (0.17, 0.8)	0.4 (0.2, 0.9)
Age of house [per 10 years increase]	0.9 (0.7, 1.0)	1.0 (0.8, 1.3)

* Adjusted for all variables shown.

Numbers may be slightly lower than the total due to missing data.

The interviews in the exposed areas were conducted between July and August 2009. The control region was interviewed in December 2009.

Volunteers also received a return envelope, a vial with ethanol, gloves and an information leaflet on kissing bugs, and were asked to send us any specimens collected from their house. They were informed about the potential risk of infection with *T. cruzi* in writing, instructed not to touch the insects, except with gloves, not to squash them and to wash their hands after handling a specimen. No specific permits were required for the described field studies, since no endangered or protected species were involved and since insects were only collected by the residents in and around their homes in residential areas.

The study was approved by the Human Research Review Board at the University of California in Riverside. All participants provided verbal consent, as specifically approved by the ethics committee. The reasons for the verbal consent were the low risks associated with the participation in the study. A standard text, which was approved by the ethics committee, was used for the introduction of the study. Consent was documented by proceeding with the interview and data collection. If the consent was refused, no data were collected.

### Statistics

Only bites and sightings, which were reported as certain, were included in the analysis. Those characterized as possible were excluded. To facilitate analysis and since they are more reliable, only symptoms (unexplained allergies, waking up) for the main interview partner were included in the analysis. Self-reported symptoms not typical for triatomine bites, e.g. “running nose”, were excluded from the analysis (n = 2). For univariate analysis, chi-square tests were used to compare categorical variables, unless expected cell sizes were less than 5, in which case Fisher Exact Test was used; T-tests were used to compare normally-distributed variables, and Wilcoxon Rank Sum Tests for non-normally distributed variables. Confidence intervals around percentage were calculated by adjusting for the clustering in sample clusters and by survey area (desert or foothills). The design effects were largely between 1 and 2 and did not exceed 3.3. To adjust for possible confounding, we used logistic regression modeling. For the adjusted model, all variables shown in Table 1 were included in the model if they were significantly associated with the outcome of interest (self-reported symptoms typical for allergies) or changed the effect estimate by more than 10%. All analyses were conducted using SAS 9.2 (Cary, NC, USA).

## Results

### Study Population

The interview teams visited 1036 houses in 20 clusters to collect 221 interviews in the exposed areas (Los Angeles County and rural area in Riverside County) (response rate 21%). This response rate did not differ from that in the Riverside County urban control area (119 of 483 houses, 25%, p = 0.15). Participants in the exposed areas did not differ in most baseline characteristics from those in the unexposed areas. Exceptions are interviewee’s sex, the age of the houses and the frequency of persons who reported leaving the light on during the night (Table 1).

### Study Outcomes

In the exposed areas 20% (95% confidence interval [CI]: 10–31%) interviewees knew about the presence of Triatominae in the neighborhood with a much higher awareness in the more rural desert area as opposed to the suburban community approaching the foothills (n = 41 [35%] versus n = 5 [5%]) (Table 2). When presented with the pinned specimens or insect images, 19% (95% CI: 11–27%) participants reported to have ever seen Triatominae in the neighborhood and 8% (95% CI: 1–14%) reported to have seen them in the house during the month before the interview. As expected, kissing bugs were most frequently sighted between dusk and midnight (n = 11 [65%]), followed by sightings around dusks (n = 10 [59%]), and between midnight and dawn (n = 9 [53%]). Only 2 (12%) persons reported sightings between noon and dusk, and 1 (6%) person at noon. No sightings were reported between dawn and noon.

The number of Triatominae seen in the house during the month before the interview was below ten with two exceptions. One interviewee reported seeing 20 and another 100 specimens. Concordant with the nightly feeding habits and painless bites of these insects [4], only 7% (95% CI: 2–12) persons reported to have ever been bitten by Triatominae.

In the control area, surveyed in winter, only 3 (3%) persons thought that Triatominae occur in the neighborhood. None had seen the insects or reported bites (Table 2).

Exposure to Triatominae was corroborated for 10 [5% (95% CI:1−8%)] study participants in the exposed areas, who collected and sent us at total of 12 specimens that we identified as *T. protracta* (7 [3%] of participants with at least one specimen ) or as *P. hirsuta* (3 [1%] persons with one or more specimens). All insects except one were collected inside homes. Most of the insects were found within a few months after the survey, three were however received as late as 15 months after the survey.

Given the possibility of unnoticed bites, we asked about unexplained allergies that matched the symptoms expected from triatomine bites [5]. Surprisingly, a large proportion (13% [95% CI:8–18%]) of the study participants in the exposed areas reported one or several of these symptoms. This frequency was 4 times higher than the value in the urban control area (29 [13%] versus 4 [3%], p = 0.005) (Table 2).

### Factors Associated with Self-reported Symptoms Characteristic of Triatomine Allergies

Persons living in the triatomine-exposed areas, who reported symptoms typical of triatomine allergies, also reported more frequently knowing about the occurrence of Triatominae in the neighborhood, having seen them more frequently, having been bitten more frequently and tended to have seen Triatominae more frequently in the last month, to be younger and to have pets more frequently than persons in the same areas who did not report any symptoms (Table 3).

### Can Self-reported Symptoms be Explained by Other Factors?

To test if the differences in self-reported symptoms between persons living in the control and the triatomine-exposed areas could be explained by underlying risk factors, we conducted multiple logistic regression modeling. When adjusted for sex and age of the house, the association of reporting symptoms typical for allergies and living in triatomine-exposed areas slightly increased (Table 4). Other variables shown in Table 1 were not significantly associated with reporting symptoms typical of triatomine allergies or changed the effect estimate appreciably.

## Discussion

This door-to-door survey in two triatomine-exposed areas and one urban control region showed that living in triatomine-exposed areas is associated with considerable contact to Triatominae and a high prevalence of self-reported allergies, which were statistically associated with exposure to Triatominae. These data therefore allow for the possibility that triatomine bites and allergic reactions are frequent in rural and suburban Southern California, which should now be further investigated using laboratory methods.

Our findings are concordant with the previous study in Santa Barbara County [10] as well as two other studies that suggested frequent exposures to Triatominae. The first study was conducted in a small rural community in Northern California and determined exposure by measuring the concentration of IgGs against insect saliva [20]. The second study asked inhabitants of the Tucson metropolitan area to collect insects [15]. To our knowledge, this is however the first study that tried to access the frequency of allergic reaction among an exposed population.

The possibility of unexplained allergic reactions to triatomine bites seems plausible, given the large likelihood of unnoticed bites [4], the here reported contact to insect exposures, the unspecific presentation of symptoms and the absence of a diagnostic test. The observation that persons with self-reported symptoms more frequently reported seeing Triatominae and been bitten than persons without those symptoms, further corroborates the possibility of a causal link between these symptoms and triatomine exposure.

Despite the plausibility of a causal link between living in triatomine-exposed areas and unexplained allergic reactions, laboratory tests are required to confirm such a link. For example, persons who reported unexplained allergies also tended to more frequently have pets than those persons who did not report these allergies. This difference in an exposure to pets as well as other unmeasured environmental variables may have contributed to the observed higher burden of unexplained allergies in triatomine exposed regions.

Another limitation of the study is that exposed sites were surveyed in summer, but the urban control in winter. An influence of seasonal effects on our results can therefore not be excluded, may have resulted in an overestimation of the association between self-reported allergies and living in triatomine exposed areas. We also cannot exclude that the definition of unexplained allergies, which was by subject understanding, was influenced by individual perception or health seeking behavior. Even though we think that these factors are unlikely to fully explain our observations, they may have contributed to the observed difference in unexplained allergies between the study areas. Furthermore, interviewers were aware of the study questions, allowing for the possibility of bias during the data collection. Finally, our data are also not representative for a large population, since we targeted areas where Triatominae were known to occur, oversampled exposed areas and had a low response-rate, possibly selecting for persons who spend more time at their house and therefore may have a higher risk for exposure.

Despite these limitations, the possibility of an unrecognized public health problem as suggested by this study, as well as the wide distribution and abundance of kissing bugs in the Western, Southwestern, and Southern United States [21], warrant further research, especially the development of commercial tests to confirm a sensiblization to triatomine bites. In addition, these data show that there is a need for a better education of the public to avoid exposure as much as possible.
